# Retrorectal schwannoma: a case report of an extremely rare location

**DOI:** 10.1093/jscr/rjaf997

**Published:** 2026-01-31

**Authors:** Jihane Zahrou, Tijani El Harroudi

**Affiliations:** Faculty of Medicine and Pharmacy, Oujda, Morocco; Department of General and Oncologic Surgery, Badr Clinic, Oujda, Morocco, 375, BD Jelloul Med Lots la Colline, Oujda 60000, Morocco

**Keywords:** retro rectal schwannoma, retroperitoneum, surgery

## Abstract

Schwannomas are tumors that arise from Schwann cells in the neural sheath. They are usually benign tumors and rarely occur in the retroperitoneal space. Retro rectal schwannomas often lack specific symptoms and present non-orienting radiologic imaging characteristics, making the diagnosis challenging. We herein report the case of a 66-year-old female patient who presented with abdominal pain and urinary retention. A presacral mass was detected on imaging and the patient underwent a surgical excision of the tumor. The diagnosis of such lesions is rigorous due to their uncommon location and very unspecific symptoms, and complete surgical resection remains the curative treatment.

## Introduction

Schwannomas are encapsulated tumors that arise from the nerve sheath of peripheral nerves. They are usually located in the head, neck or the upper extremities, although they may appear in the posterior mediastinum and, even more unlikely, in the retroperitoneum [1]. Retro rectal schwannoma is usually a benign tumor that is very infrequent and presents with non-specific symptoms. At most, ~3% of Schwannomas are located in the retroperitoneum, and they predominantly occur in women [2]. We herein report the case of an atypical location of Schwannoma in a 66-year-old female patient who presented with abdominal pain and urinary retention.

## Case report

A 66-year-old woman with a history of diabetes mellitus presented with severe abdominal pain and urinary retention. On clinical examination, she appeared in stable condition, and initial investigations revealed normal blood counts and serum CA-125 levels. Renal function tests were unremarkable, aside from mild renal insufficiency as per the MDRD formula.

Abdominopelvic computed tomography revealed a a well-circumscribed, hypodense pelvic mass measuring ~110 × 138 mm. Right ureter and right renal pelvis were dilated with no visible obstacle.

**Figure 1:**
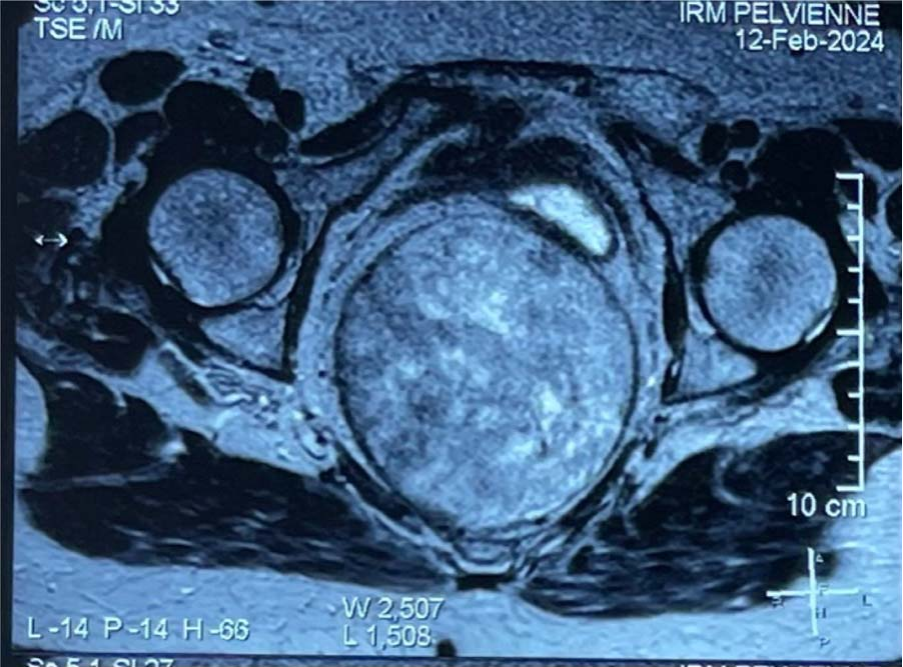
Pelvic MRI of the mass.

Pelvic magnetic resonance imaging (MRI) showed a voluminous, 12.5 × 10.5 cm, retro rectal mass exerting anterior compression on the rectum, displacing it laterally (Fig. 1).

Rectosigmoidoscopy revealed an extrinsic mass effect on the posterior rectal wall. Histopathological examination of rectal biopsies yielded non-conclusive results.

Abdominal ultrasound further confirmed bilateral hydronephrosis secondary to extrinsic compression from the pelvic mass.

**Figure 2:**
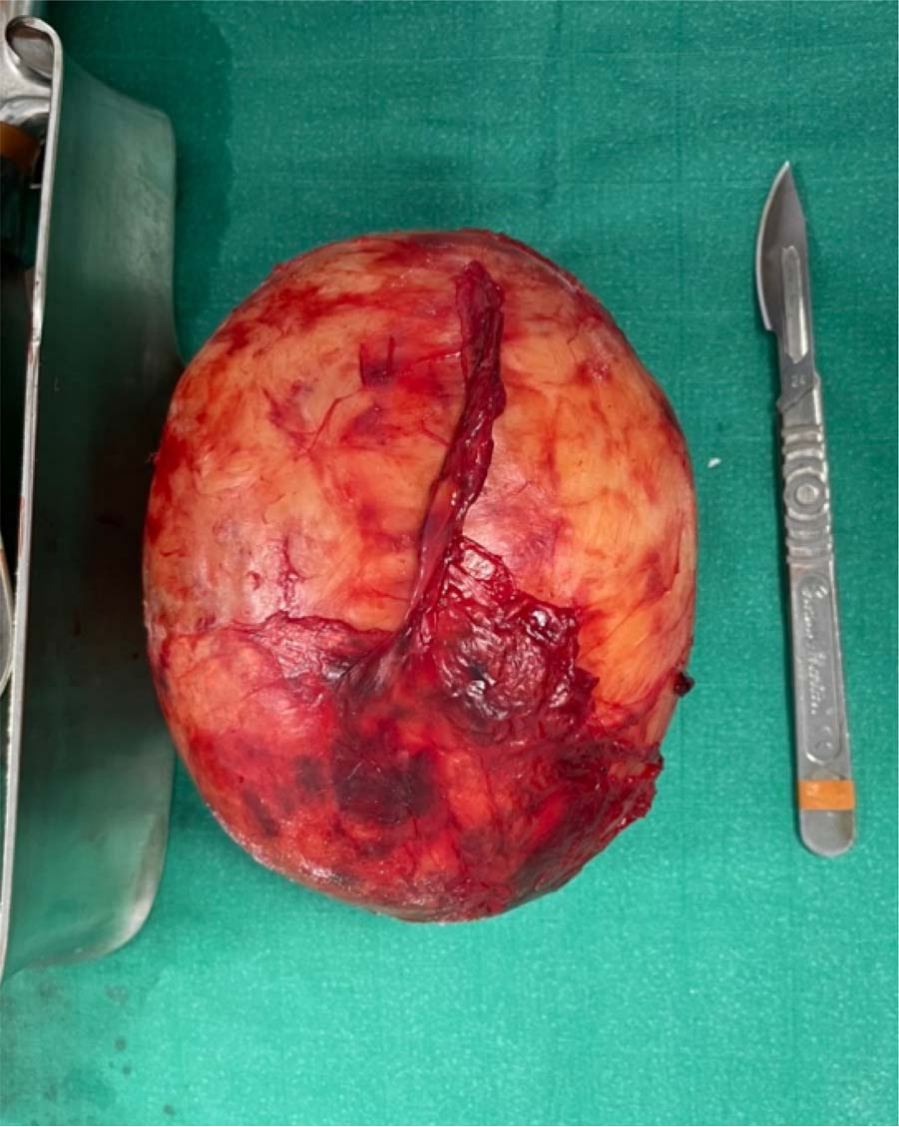
The operative specimen.

Given these findings, the patient underwent surgical excision under general anesthesia via an abdominal approach through a lower midline incision. Intraoperatively, the rectum was mobilized, revealing a well-defined, voluminous retro rectalmass in close proximity to the right iliac artery and vein. The lesion was meticulously dissected and completely excised from the retroperitoneum. Macroscopic and perioperative examination revealed a solid, ovoid tumor encapsulated by a smooth capsule (Fig. 2).

Histopathology demonstrated proliferation of spindle-shaped cells arranged in palisading patterns characteristic of Antoni A regions, intermixed with looser Antoni B areas. The tumor was vascularized by thick-walled, hyalinized vessels, and inflammatory infiltrates were present. These features were highly suggestive of schwannoma. Immunohistochemical analysis revealed strong positivity for S100 protein and smooth muscle actin, while staining negative for desmin and CD34, confirming the diagnosis. The postoperative course was uneventful, and the patient was discharged in stable condition.

## Discussion

Schwannomas, or neurilemmomas, are benign, encapsulated tumors originating from Schwann cells of peripheral nerve sheaths [1]. While frequently encountered in the head, neck, and upper limbs, retroperitoneal schwannomas are rare, accounting for ~3% of cases, predominantly affecting females aged 20–50 years [2]. The first documented retroperitoneal schwannoma was reported in 1944 by Stallworthy [3].

The retrorectal space, defined anatomically as the region between the rectum anteriorly and the sacrum posteriorly, [4] is bounded by the rectal fascia anteriorly, Waldeyer’s fascia posteriorly, and the iliac vessels and ureters laterally [5]. Its expansive, non-restrictive nature often permits tumors to attain substantial size before symptom onset. Consequently, clinical presentation is often insidious, with nonspecific symptoms such as lower back pain, renal colic, or vague abdominal discomfort, frequently leading to delayed diagnosis [1]. Middle-aged patients with chronic lower back pain are frequently misdiagnosed, further postponing diagnosis and treatment [2]. Our patient presented with vague symptoms complicated by bilateral hydronephrosis secondary to extrinsic ureteral compression.

Macroscopically, schwannomas appear as solitary, firm, well-circumscribed masses, sometimes exhibiting degenerative changes like hemorrhage or calcification [3].

**Figure 3:**
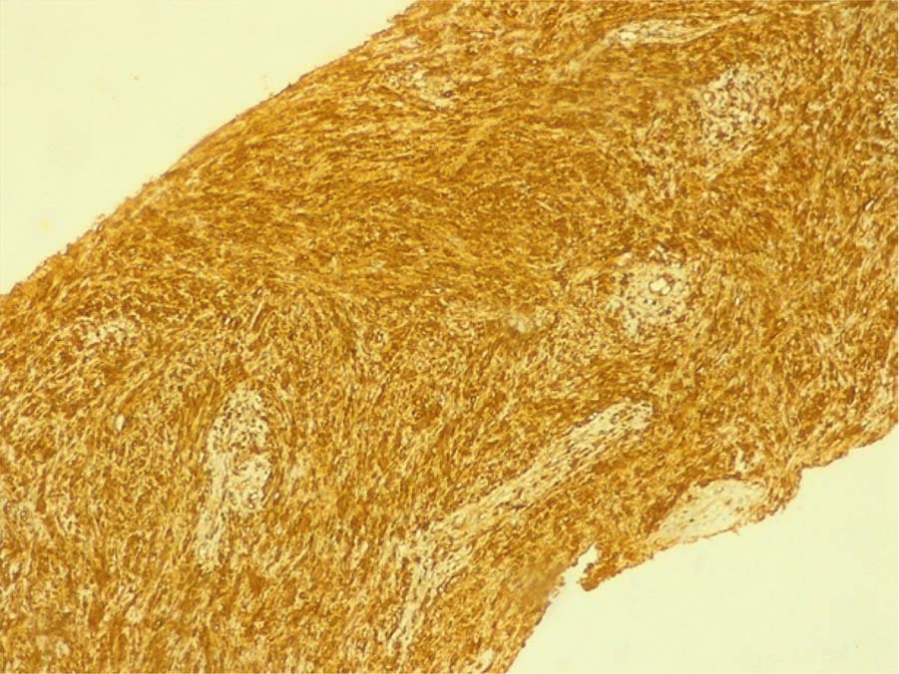
The immunohistochemistry findings.

**Figure 4:**
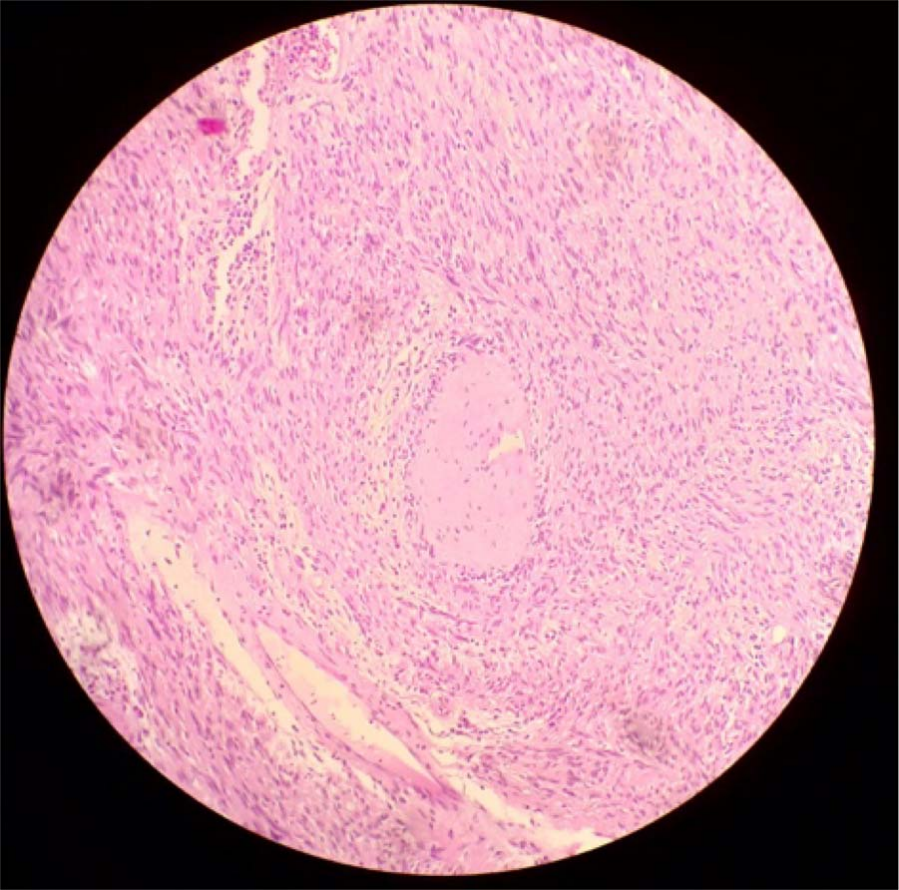
Microscopic histopathological examination.

Microscopically, the tumor is made out of elongated cells arranged in alignments known as palisades that take one of two patterns: Cells in Antoni type A are displayed in an arranged and firm pattern, while cells in Antoni type B are dispersed loosely within an edematous matrix (Figs 3 and 4). Both of these patterns can coexist and malignant changes are rare [6]. Definite diagnosis is made through histopathological examination and immunohistochemistry [1]. S100 protein positivity and vimentin present hallmark of schwannoma [2].

In our case, histopathological findings showed spindle cell proliferation with Antoni A and Antoni B regions, with the presence of S100 protein and vimentin in the immunohistochemical study.

Radiologically, computed tomography (CT) scans and MRI are the two key exams of evaluating sacral masses and planning the operative approach [7]. CT scans usually reveal a smooth, well-defined mass with soft-tissue density. MRI is currently considered as the imaging method of choice since it provides superior tissue characterization, such as solid tissue, fibrous tissue, simple or atypical fluid, and blood [1]. Schwannomas generally exhibit low signal intensity on T1-weighted images and high signal intensity on T2-weighted images, influenced by Antoni tissue patterns. These findings may be characteristic, however they are not specific enough to make the preoperative diagnosis of retroperitoneal schwannoma [3]. Differential diagnoses include fibrosarcoma, liposarcoma, and ganglioneuroma. All these lesions have analogous findings and can be challenging to distinguish radiologically, which makes the preoperative diagnosis rather challenging [3].

In our case, imaging confirmed the presence of a well-defined, voluminous, retro rectal mass, hypo and hyperintense on T2-weighted images and hypointense on T1-weighted images.

Complete surgical excision remains the treatment of choice, given schwannomas’ resistance to radiation and chemotherapy [2]. Laparoscopic excision and robotic resection emerge as promising surgical approaches, however the location and the size of tumors may dictate the choice of surgical approaches [3]. During tumor dissection, heavy hemorrhage may be encountered from the vessels nearby, this may require massive blood transfusion [6].

Our case involved open abdominal surgery via a lower midline incision as a result of the limited space and close relationship with surrounding organs and vessels. The tumor was successfully excised with preservation of surrounding structures. Histopathology confirmed schwannoma, aligning with imaging and intraoperative findings.
